# Memory complaints after COVID‐19: a potential indicator of primary cognitive impairment or a correlate of psychiatric symptoms?

**DOI:** 10.1002/alz.094786

**Published:** 2025-01-09

**Authors:** Yiling Dong, Ana Paula Ritto, Rodolfo Furlan Damiano, Amanda Goulart Coli, Rodrigo Hadade, Cristiana Castanho de Almeida Rocca, Antonio de Pádua Serafim, Bruno Fukelmann Guedes, Ricardo Nitrini, Orestes Vicente Forlenza, Geraldo Busatto Filho, HCFMUSP COVID‐19 Study Group

**Affiliations:** ^1^ The George Washington School of Medicine and Health Sciences, Washington, DC USA; ^2^ Hospital das Clínicas da Faculdade de Medicina da Universidade de São Paulo, São Paulo, São Paulo Brazil

## Abstract

Cognitive impairment and symptoms of psychiatric disorders have been reported frequently as features of post‐acute sequelae of SARS‐CoV‐2 infection. This study aims to investigate subjective memory complaints in COVID‐19 survivors and determine if these are more strongly associated with objective cognitive impairment related to sequelae of SARS‐CoV‐2 infection or with symptoms of psychiatric conditions. A total of 608 COVID‐19 survivors were evaluated in‐person 6 to 11 months after hospitalization, with 377 patients assigned to a “no SMC” group and 231 patients assigned to an SMC group based on their Memory Complaint Scale scores. Follow‐up evaluations included an objective cognitive battery and scale‐based assessments of anxiety, depression, and post‐traumatic stress symptoms. We found the perception of memory impairment in COVID‐19 survivors to be more strongly associated to core symptoms of psychiatric conditions rather than to primary objective cognitive impairment. Univariate analysis indicated significant differences between the “no SMC” and SMC groups, both for the psychiatric symptom evaluations and for the cognitive evaluations (p<0.05); however, the psychiatric symptoms all had large eta‐squared values (ranging from 0.181 to 0.213), whereas the cognitive variables had small/medium eta‐squared values (ranging from 0.002 to 0.024). Additionally, multiple regression analysis indicated that only female sex and depressive and post‐traumatic stress symptoms were found to be predictors of subjective memory complaints. These findings may help guide clinical evaluations for COVID‐19 survivors presenting with memory complaints while also serving to expand our growing understanding of the relationship between COVID‐19, subjective memory complaints, and the risk of cognitive decline.

